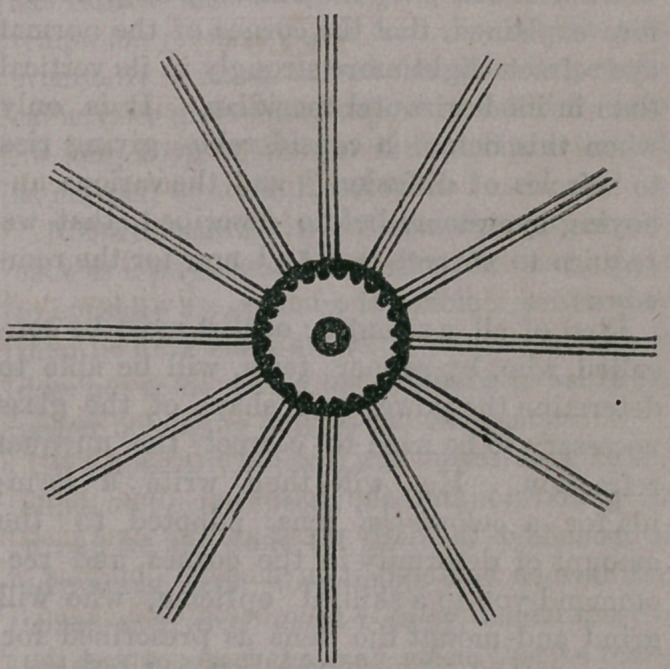# Astigmatism

**Published:** 1872-04

**Authors:** 


### ASTIGMATISM.

By astigmatism we mean that peculiar condition of the eye by which the light is unequally refracted, giving rise to indistinctness of vision. It depends upon a variation in the curvature of the two meridians of the cornea, causing the rays of light passing from an object through the vertical meridian, to be sooner refracted than those traversing the horizontal meridian, or *vice versa*.

By the meridian of the eye we are understood to mean, that if the axis of the eye be represented by an imaginary straight line, passing directly through the centre of the cornea to the retina, the plane that would intersect this axis vertically, would be termed the "vertical meridian," while that which crosses it horizontally, is called the "horizontal meridian." Distinct vision requires that the rays of light emanating from an object under observation, should focus accurately upon the retina. Should the refraction be greater in the vertical meridian than in the horizontal, the rays passing through the former are brought to a focus at a shorter distance from the cornea than the latter, giving rise to what is termed by the oculist "circles of diffusion," or a number of indistinct images which blur the principal one.

Astigmatism is often congenital, though it is frequently produced through inflammation or ulceration of the cornea, through which its shape becomes altered, giving rise to the irregular refraction of light from different points of its surface. Blows upon the eye produce astigmatism often, by slightly dislodging the crystalline lens, thus changing its axis. It may also exist in one eye while the opposite one remains normal, though it is most frequently found in both eyes.

Persons having this defect of the eyes observe that they see horizontal lines more clearly than vertical ones, or the reverse. Points appear as lines, circles as ovals, and squares as rectangles. A circular hole cut through a card-board will appear oval, and if made square will seem rectangular.

The defect in vision produced by even a slight degree of astigmatism, is often extremely annoying, especially in reading; the shape of the letters being so changed as to render it very difficult to distinguish them without constantly varying the distance between the book and the eye. The letters appear blurred, some of their lines being perfectly distinct, while others cannot be discerned at all. Thus, the vertical lines of the letter H may appear quite clear, while the horizontal, connecting line, is quite invisible, giving the letter the appearance of I I. Often persons having this defect of the eye are not conscious of it, their attention never having been called to these defects of form. All they know is that their vision is very indistinct, and that so much discomfort is caused by an attempt to read or sew, that it is rarely they engage in such employments.

In order to enable persons afflicted with indistinct vision to decide whether they have astigmatism or not, we have caused the following engraving to be made, by means of which the infirmity can be readily detected:

In holding the magazine directly before your eyes, about thirteen inches from the face, if you can see the three black lines, with the two white spaces between, equally distinct,—the lines appearing quite as black, and the spaces regular, and clearly defined, in every direction of the spokes in the wheel; and if the circles in the centre are round and not oblong, you are not troubled with unequal refraction, or astigmatism. Upon the contrary, if you find the vertical lines to appear darker or longer, or that the white spaces are more clearly seen in this direction than those that run horizontally or obliquely, or *vice versa*; or if by turning the engraving slowly before your eyes you find the horizontal lines become more distinct,—appearing blacker and clearer for being made vertical, you are a victim of astigmatism.

Be it understood, however, that *all* eyes are slightly astigmatic, for in the perfectly normal eye the vertical meridian has a shorter focal distance than the horizontal. This will be verified in again examining our engraving, when we will find that we are unable to distinguish both the horizontal and vertical lines with equal distinctness, at one and the same time. It will be found that when we see the vertical lines clearly, the horizontal ones must be brought a trifle nearer to the eye, before they are equally distinct. It has doubtless been noticed by many of our readers that a vertical stripe can be seen much further off, than a horizontal one,—that the vertical piece in a cross, for instance, can be seen at a greater distance than the horizontal bar; the reason being, as before explained, that the cornea of the normal eye refracts light more strongly in its vertical than in its horizontal meridian. It is only when this defect is considerable, giving rise to "circles of diffusion," and the various annoying symptoms before described, that we require to correct it. And now for the remedy:

First of all, a scientific oculist must be consulted, who, by proper tests, will be able to determine the power and shape of the glass necessary to be used to correct the unequal refraction. He will then write a formula for a *cylindrical* lens, adapted to the amount of deformity in the cornea, and recommend you to a skillful optician, who will grind and mount the lens as prescribed for your particular case. You will find it impossible to select your own glasses, or to have a jeweler or spectacle vender do it for you. Ordinary near or far-sighted glasses will not answer the purpose; you can try on every lens in your jeweler's establishment, without being able to find a pair that will improve your vision in the least. *Nothiny but cylindrical lenses, properly ground and adjusted, will afford relief.* By a cylindrical lens, we mean one that is a segment of a cylinder, and *not* of a sphere, as is the case with the usual glasses used for near or far sightedness. The first mentioned lens refracts those rays the strongest which strike it at right angles to the axis of curvature, while those which pass through its centre suffer no deviation; the spherical or ordinary lens refracts the rays in all planes of the segment, being therefore of no service in astigmatism.

Our object in writing this series of articles upon the eye, is to give our readers some general ideas with reference to the most common defects of vision, and to teach them that it is always best to consult a skillful ophthalmic surgeon for almost any ailment of the eyes, rather than to employ patent nostrums, for sale in the shops, or to receive treatment from the traveling quacks who infest almost every neighborhood. Another fact to be learned by the people is, that spectacles should *never* be employed without the advice and consent of a competent surgeon. Jewelers and traveling spectacle venders are not competent to decide when glasses are necessary, nor the sort to be used. Hundreds of people ruin their eyes with glasses not adapted to their cases, and thousands are suffering from imperfect vision, not knowing that properly constructed lenses would afford them permanent relief.

## Figures and Tables

**Figure f1:**